# Viroimmunotherapy of Thoracic Cancers

**DOI:** 10.3390/biomedicines5010002

**Published:** 2017-01-04

**Authors:** Alexander S. Dash, Manish R. Patel

**Affiliations:** 1 Department of Biology, Macalester College, St. Paul, MN 55105, USA; adash@macalester.edu; 2 Division of Hematology, Oncology, and Transplantation, University of Minnesota Medical School, Minneapolis, MN 55455, USA

**Keywords:** oncolytic virus, thoracic cancers, lung cancer, mesothelioma, immunotherapy, viroimmunotherapy, immunogenic cell death, immune infiltration

## Abstract

Thoracic cancers, including non-small cell lung cancer (NSCLC), small cell lung cancer (SCLC), and malignant pleural mesothelioma (MM), cause the highest rate of cancer mortality worldwide. Most of these deaths are as a result of NSCLC; however, prognoses for the other two diseases remain as some of the poorest of any cancers. Recent advances in immunotherapy, specifically immune checkpoint inhibitors, have begun to help a small population of patients with advanced lung cancer. People who respond to these immune therapies generally have a durable response and many see dramatic decreases in their disease. However, response to immune therapies remains relatively low. Therefore, intense research is now underway to rationally develop combination therapies to expand the range of patients who will respond to and benefit from immune therapy. One promising approach is with oncolytic viruses. These oncolytic viruses (OVs) have been found to be selective for or have been engineered to preferentially infect and kill cancer cells. In pre-clinical models of different thoracic cancers, it has been found that these viruses can induce immunogenic cell death, increase the number of immune mediators brought into the tumor microenvironment and broaden the neoantigen-specific T cell response. We will review here the literature regarding the application of virotherapy toward augmenting immune responses in thoracic cancers.

## 1. Introduction

Cancers of the thorax, including non-small cell lung cancer (NSCLC), small cell lung cancer (SCLC), and mesothelioma (MM), cause the highest rate of cancer-related mortality worldwide [1]. The bulk of these fatalities are attributed to non-small cell lung cancer, but this fact belies the devastating toll the latter two diseases impart on afflicted patients. The vast majority of patients are not diagnosed until their disease is incurable. Though advances have been made in NSCLC, median survival remains poor, and there has been little to no improvement in outcomes for MM or SCLC over the past two decades.

Immunotherapy, using anti-programmed death 1 (anti-PD1) antibodies, has recently been approved by the Food and Drug Administration (FDA) for NSCLC based on a statistically significant improvement in survival compared to standard chemotherapy [2]. For SCLC and MM, data has been presented to suggest that anti-PD1 antibodies may be as effective as chemotherapy for these diseases as well [3,4]. While there are significant differences among these disease types, within each disease, there is a subset of patients who can have both dramatic and durable antitumor responses. Unfortunately, the response rates remain low, in the range of 15%–30%. Thus, there are now ongoing efforts to improve upon these results using novel combination therapies. Importantly, after nearly 40 years of promising to develop immune therapy for cancer, research has finally developed a breakthrough that is applicable to a variety of common and devastating malignancies, providing hope for a cure.

A new and emerging field in the realm of immunotherapy is the use of oncolytic viruses [5]. These viruses cause both direct lysis of tumor cells as well as induce an enhanced immune response to the cancerous tissue. Genetic engineering has allowed the development of recombinant viruses that can express immunotherapeutic cytokines, amplifying their antitumor activity and increasing their specificity for heavily mutated tumor cells. Numerous studies have now demonstrated that several oncolytic viruses have immunomodulatory effects favoring antitumor immunity [6,7,8,9]. Since immunotherapy is now a part of standard therapy for NSCLC, we will review here the application of virotherapy as a potential immunotherapy when applied to lung cancer and mesothelioma. Furthermore, we will examine what is known about the mechanisms involved and potential strategies for rational viral combination therapies.

### Application of Virotherapy for Thoracic Cancers

As a pathogen, viruses are naturally immunogenic and the host has evolved several innate and adaptive immune mechanisms to limit viral spread and prevent reinfection with the same virus. Since many of the oncolytic viruses are common pathogens, most people have been previously exposed to these viruses and have circulating neutralizing antibodies. Even for the few animal viruses that are used, neutralizing antibodies will develop. This creates a major limitation to oncolytic virotherapy systemically as circulating virus can be rapidly cleared by neutralizing antibodies, preventing the virus from ever reaching the tumor to exert its effects. As a result, pleural mesothelioma seems to be an ideal candidate for virotherapy as the vast majority of patients have disease confined to one hemithorax and the pleural space is accessible via a catheter. Furthermore, work by several investigators has demonstrated that the immune repertoire in the pleural space may be important for disease control and can be modulated by viral gene therapy delivered to the pleural space [10,11]. On the other hand, lung cancer (either NSCLC or SCLC) is a systemic disease hallmarked by early hematogenous metastasis, and thus pre-existing neutralizing antibodies are problematic for systemic delivery and local delivery could be difficult and may not address the systemic tumor burden. The finding that talimogene laherparepvec (Tvec), a recombinant herpes simplex virus expressing granulocyte-monocyte colony-stimulating factor (GM-CSF), was able to evoke a systemic immunotherapeutic response after local injection of dermal melanoma lesions challenged the notion that local tumor injection could not be efficacious for widespread disease [6]. This study has led to the first FDA-approved oncolytic virus in the United States. These developments have led to the possibility of similar approaches for the treatment of NSCLC. Though not as simple as intradermal injections, NSCLC tumors are often accessible using endobronchial approaches with ultrasound (EBUS) guidance directly into diseased mediastinal lymph nodes or endobronchial tumors for potential viroimmunotherapy [12]. This article will review the pertinent literature to date using oncolytic viruses to achieve enhanced antitumor immune responses in thoracic malignancies. These studies demonstrate that the stage is now set to advance oncolytic viroimmunotherapy to the clinical setting for thoracic malignancies.

## 2. Oncolytic Viruses for Thoracic Cancers

Currently, there are numerous agents receiving attention as a potential viral immunotherapy for thoracic cancers. A few of these include the vesicular stomatitis virus (VSV), measles virus (MV), vaccinia virus (VV), and adenovirus (Ad) (Table 1). A small number of these viruses have made it into the clinic (Table 2) where they are being tested in conjunction with various other drugs to see how they can improve outcomes for patients with incurable thoracic cancers. Reovirus is one of the few trials that has been published in patients with NSCLC. This phase II trial combined reovirus with standard chemotherapy in patients with activation of the epidermal growth factor pathway. This patient population was chosen as the oncolytic activity of reovirus depends upon signaling through the Kras pathway and inhibition of protein kinase to double-stranded RNA (PKR) [5]. The objective response rate was 31%, which is higher than what is expected with chemotherapy alone; however, the lack of a comparator arm limits any conclusions that could be drawn from this trial [13]. These results do align with preclinical data suggesting that reovirus is synergistic in combination with chemotherapy for NSCLC [14].

Seneca valley virus (SVV) was initially found to be tropic for neuroendocrine tumors. While the mechanism of the tumor tropism of SVV is not clear, in the initial phase I trial, one patient with SCLC had prolonged stable disease for longer than 10 months. Thus, patients with SCLC who had completed induction chemotherapy were randomized to receive SVV-001 or a placebo in a randomized phase II trial. Though the results were not published, the data were presented and found no benefit of SVV-001 in this setting. There was no signal of activity whatsoever. Thus, further development of SVV for small cell lung cancer has been abandoned. Several other trials are under way or completed; however, published results are not yet available. The primary endpoint for most of the trials involves the safety and feasibility of the use of oncolytic viruses in humans. Many of these pathogens are being investigated as combination therapies to see how they can augment the efficacy of already established treatment regimens as well as complement other novel biologic and immunologic agents currently being studied. While the primary endpoint for most of these trials is simply the safety profile, there are also plans to test for immunological markers in the tumor microenvironment which could indicate if viroimmunotherapy increases tumor infiltration by T cells, shows decreases in the presence of immune suppression cells, and increases the humoral immune response.

Many of these viruses have been further attenuated through genetic modification in order to enhance viral replication in tumor cells and improve the safety profile in healthy cells. One common modification is the addition of interferon-β (IFNβ) [15,16]. IFNβ is the key innate mechanism to inhibiting viral replication in healthy human cells. There are often defects in the type I IFN response of many tumor tissues allowing for increased viral replication, leaving normal cells unaffected. While this modification has been pursued to generate increased tumor specificity and safety, it is also clear that IFNβ can play a role in stimulating immune responses. For lung cancer with malignant pleural effusion and mesothelioma, gene therapy using a replication-deficient adenovirus engineered to express IFNβ has been tested in a phase I clinical trial by direct instillation into the pleural space carried by a non-replicating adenoviral vector. Though clinical responses were rare, some responses did occur. Moreover, there was evidence of stimulation of antitumor immune responses in seven of 10 patients [10]. The lack of clinical response was felt to be limited by the low infection efficiency and, thus, the low levels of produced IFNβ. Thus, viral spread of a replication competent virus could increase the exposure of the tumor microenvironment to this immune stimulus.

Thus, several viruses have been engineered to produce IFNβ that have been applied to thoracic cancers. Measles, vaccinia, and vesicular stomatitis virus have all been engineered to produce IFNβ and have been tested in laboratory models of NSCLC or mesothelioma with profound antitumor effects in mouse models [8,15,17]. As yet, none of these viruses have been tested in clinical trials. Interestingly, though conditionally replicative adenovirus has been engineered to produce IFN, it has not yet been tested in NSCLC or mesothelioma models to date [18]. Though a variety of recombinant viral vectors have been engineered to stimulate the immune system, relatively few have been applied to thoracic cancers as of yet. With the FDA approval of immune checkpoint inhibitors for NSCLC, it is likely that further development of these novel viral vectors will be applied towards thoracic malignancies in the near future.

There are also currently numerous clinical trials being investigated at institutions across the world.

## 3. Mechanisms of Viroimmunotherapy in Thoracic Malignancies

### 3.1. Immunogenic Cell Death

As an oncolytic agent, viruses directly lyse cancer cells. It is likely that this action of oncolytic viruses can directly stimulate antitumor immune responses. Viral infection involves the creation of viral RNA and or DNA within the cytosol, which sends a danger signal from the infected cell. Though there are several pathways that are involved in this danger signal, the end result involves the release into the tumor microenvironment of damage-associated molecular patterns (DAMPS) and pathogen-associated molecular patterns (PAMPS) by the dying cell which can trigger an immune response [7,35]. Though not all cytotoxic agents induce these signals, oncolytic viruses do. In the case of NSCLC, coxackie b virus, a single-stranded RNA virus, has been shown to have profound oncolytic effects [22]. Perhaps one of the major mechanisms involves the inducement of immunogenic cell death with robust production of extracellular ATP, high mobility group box 1 (HMGB1), and cell-surface expression of calreticulin, markers of immunogenic cell death [35]. Moreover, intratumoral injections led to marked tumor infiltration with granulocytes, activated dendritic cells and NK cells. Depleting experiments demonstrated that the antitumor activity was dependent upon NK cells and granulocytes, highlighting the importance of innate immune stimulation in mediating antitumor activity.

Measles virus has been found to be oncolytic for both NSCLC and mesothelioma. Unfortunately, the MV host range does not include mice, and therefore there is no immune-competent model to effectively study MV viroimmunotherapy. However, there are some data to suggest that MV can induce immunity in mesothelioma. MV-infected mesothelioma cells resulted in activation of human dendritic cells and cross-presentation to mesothelioma-specific T cells in co-culture experiments [26]. Dendritic cell maturation and induction of tumor-specific T cells were dependent on the viral infection of mesothelioma cells as UV irradiation did not induce the same response. It is likely that the activation of the pattern recognition receptors, toll-like receptor 3 (TLR3), RIG-1, and MDA-5, after viral infection mediates this response, though this has not been carefully elucidated in this model. Both of the above instances highlight the effects of viral oncolysis on stimulating innate immune responses in the tumor microenvironment.

### 3.2. Broadening of the Spectrum of Neoantigen-Specific T Cell Responses

Beyond simply oncolysis, the cell death associated with oncolytic virotherapy may involve additional mechanisms that serve to evoke an adaptive immune response. One reason that NSCLC is immunogenic is the fact that there are typically numerous nonsynonymous mutations present within any individual tumor [36]. These resulting proteins are potentially immunogenic and can be presented on Class I MHC molecules. The presence of numerous mutations increases the likelihood of recognition by tumor-specific T cells. Viral oncolysis can activate signals that lead to enhanced presentation of these neoepitopes on MHC molecules and can thusly expose tumor antigens to the immune system that had previously gone unnoticed [31]. It has been shown using a murine NSCLC cell line that replicative adenovirus infection can broaden the antitumor immune response by increasing the number of tumor mutations recognized by tumor-specific T cells [20]. Neither untreated tumor-bearing mice nor mice treated with immune checkpoint blockade were able evoke this response. Moreover, synthetic ligands of toll-like receptors (TLRs), CpG and poly(I:C) were unable to evoke this epitope-spreading phenomenon, suggesting that the viral replication is in some way important in mediating this effect, though the mechanism has not been fully elucidated. This data reveals a potential mechanism by which viral oncolysis can increase the chance of immune recognition by a tumor-specific T cell and lead to antitumor immune responses. In addition, combination therapy with immune checkpoint blockade seems to be a rational strategy based on these results as the response to therapy correlates with the tumor mutational burden and immune recognition of the resulting neoepitopes. Several groups are now considering such trials combining oncolytic virotherapy with immune checkpoint blockade specifically for NSCLC. It will be important to study these potential mechanisms in parallel with the clinical trial to better understand the critical mechanisms of response in combination with oncolytic virotherapy.

### 3.3. Recruitment of Immune Mediators to the Tumor Microenvironment

As a pathogen, oncolytic viruses in the tumor microenvironment provide a potent stimulus to attract infiltrating immune cells to the tumor microenvironment. In models of NSCLC and mesothelioma as well as many other tumor types, this outcome is consistent and reproducible. While undoubtedly much of the infiltrate is directed at the virus, this inflammation can be beneficial. Inflammation created by viral infection at the tumor microenvironment can create a bystander effect in which activated NK cells and dendritic cells can be alerted to the presence of tumor cells. Moreover, the inflammation can result in the secretion of chemokines (IL-10, e.g.), which can attract further T cells, some of which undoubtedly are tumor-specific. Intratumoral VSV expressing IFNβ (VSV-IFNβ) results in a profound change in the tumor microenvironment of murine lung cancer [8]. There is a complete reversal of the immune suppression seen with a marked reduction in immune-suppressive T regulatory cells (Tregs) and myeloid-derived suppressor cells (MDSC) and an increase in both CD8 T cells and effector CD4 T cells in conjunction with tumor-infiltration leukocytes (TILs). Importantly, this change is not limited to the injected tumor, but rather is systemic. Uninjected tumors show almost identical effects with regards to immune infiltration after virotherapy. VSV-IFNβ has been tested in murine mesothelioma models as well [16]. In this case, the immune infiltrate was important as CD8 depletion resulted in abrogation of antitumor activity. Similarly, vaccinia virus expressing IFNβ is oncolytic for murine lung cancer [15]. Interestingly, the mechanism of antitumor activity was distinct depending on which murine model was used. In one model, TC-1, the therapeutic efficacy was dependent upon robust viral replication, whereas in the other LKRM2 model, viral replication was minimal and the response was entirely dependent upon immune activation as the antitumor activity was greatly inhibited when tumors were grown in immune-deficient mice and in CD8 T cell-depleted mice. Notably, a vaccination strategy in the TC-1 model (HPV E7-driven cancer) was much more effective in combination with VV-IFNβ, which was dependent on the IFNβ transgene, highlighting the potential for viroimmunotherapy combinations.

The immune infiltration may, in part, be dependent upon the expression of the IFNβ transgene. VV-IFNβ induced CXCL10 production compared to VV-Luc in the above NSCLC mouse models. In contrast, MV did not induce CXCL10 production when co-cultured with human dendritic cells in the mesothelioma model [26]. MV engineered to express IFNβ did induce two- to four-fold higher infiltration of CD68 monocytes in athymic mice bearing mesothelioma tumors compared to parent virus, demonstrating that the IFNβ transgene may play a therapeutic role in stimulating innate immune responses in the tumor microenvironment; however, CXCL10 production was not assayed [17]. Still since CXCL10 is known to play a critical role in attracting T cells into the tumor microenvironment and is under the control of type I IFN, it seems reasonable to speculate that the IFNβ transgene modulates the chemokine milieu to recruit immune effectors into the tumor microenvironment. Undoubtedly, the production of such chemokines will be tumor- and context-specific and may be an important biomarker for determining outcomes after virotherapy.

## 4. Discussion and Future Directions

**Figure 1:**
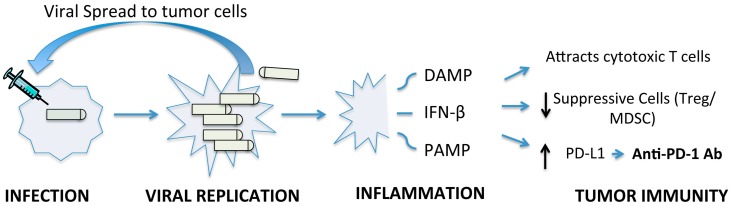
Schematic representation of possible mechanisms by which oncolytic virotherapy can exert immunologic effects in the tumor microenvironment. Initial viral infection of the tumor cell leads to viral replication and lysis. Progeny virions then can infect and lyse surrounding cells amplifying tumor lysis. This infection can then directly lead to release of damage associated and pathogen associated molecular patterns (DAMP and PAMP, respectively) as well as interferons into the tumor microenvironment. These can further attract T cells, decrease suppressive cells, and lead to upregulation of programmed death ligand (PDL-1) on tumor cells promoting antitumor immunity.

For decades, there has been interest in the natural tropism of viruses for cancer cells and direct oncolytic activity. There has been a recent paradigm shift in the therapeutic use of viruses as a cancer immunotherapy with emerging data demonstrating the dependency of immune stimulation on the improved outcomes in various cancer models (Figure 1). Furthermore, initial fears about the safety of live viral vectors have given way to the exciting promise that these treatments hold. The emergence of immune checkpoint blockade for thoracic malignancies has now paved the way for novel combination therapies. While anti-PD1 antibodies have demonstrated improvement in outcomes for patients with thoracic malignancies, the response rate remains low. Data in melanoma and NSCLC indicate that pre-existing T cells in the tumor microenvironment and programmed death ligand (PDL)-1 expression on the tumor are associated with response to therapy. Furthermore, in spite of high PDL-1 expression, tumors without infiltrating T cells are unlikely to respond. We and others have shown that oncolytic virotherapy has the potential to recreate the tumor microenvironment needed for response to anti-PD1 antibodies. The promise of this approach has already been published with adenovirus in the NSCLC model and several groups are actively pursuing this approach for clinical translation.

Going forward there will be several issues that remain to be addressed with regards to clinical application of viroimmunotherapy. First, viral delivery remains a thorny issue for some viruses. Vaccinia and reovirus are good viruses for systemic delivery; however, the doses required to achieve systemic tumor replication are quite high and near the limits of production capacity. While we have argued the case for intratumoral delivery, it is both expensive and inconvenient. Therefore, strategies for effective targeting or evasion from systemic immunity remain relevant for maximizing the ability to apply virotherapy for patient care, particularly for patients with advanced lung cancer. The use of cellular carriers of MV is already entering clinical testing for ovarian cancer [37]. The results of these studies and several other ongoing efforts to develop strategies for systemic administration of oncolytic viruses are eagerly awaited.

Another major issue is the complex interaction between the host, tumor, and pathogen which can have a drastic impact on outcomes. It is clear that some tumors are exquisitely sensitive to viral replication and others are not. Similarly, it is likely that some tumors are very sensitive to immune therapy while others are not, and there is likely to be significant overlap between the viral-sensitive and the immune therapy-sensitive tumors. For combination studies, the effects of immune stimulation on viral replication may be deleterious for the viral-sensitive type of tumors. Therefore, careful experiments to understand the optimal timing of immune therapy in the context of oncolytic virotherapy will be important. Moreover, given the substantial differences in immunity and tumors between mouse and human, it will be paramount to study the changes in the tumor microenvironment before and after virotherapy during clinical testing. Careful design of clinical trials with the goal of understanding treatment response and resistance will guide the development of the next generation of viral vectors and better deployment of the ones we already have.

There is no question that we have entered a new and exciting phase of development of oncolytic viruses as an immunotherapy for cancer. The ability of viruses to be directly cytotoxic to cancer cells offers a distinct advantage over other immune therapy strategies that rely on indirect immune stimulation. Unlike other therapies for thoracic cancers, immune therapy with checkpoint blockade has not only been generally well-tolerated, but also tantalizing in the offer of durability of response. Currently, there is an intense effort at finding effective combinations of checkpoint blockade with other immune therapy with the hopes of increasing the response rate such that the majority of patients can receive the durable benefit that so far only applies to 15%–30% of the patient population. Preclinical data with oncolytic virotherapy suggest that viruses can play an important role as an immunotherapy in their own right, but also in combination with checkpoint blockade. The time has come for clinical testing for thoracic cancers and results of upcoming early-phase trials are eagerly awaited.

## Figures and Tables

**Table 1:** Oncolytic viruses that have been studied in thoracic cancers.

Oncolytic Virus	Disease	Immune Mechanism Studied	Phase of Development	Author/Year/Journal
Adenovirus	NSCLC	N/A	Pre-Clinical Data: Nude Mice	Zhang, J.-F., et al., 2010. [19].
	NSCLC	Neoantigen Specific Response; Checkpoint-Inhibitors; CD8+ Tumor Infiltration	Pre-Clinical Data: Immune Competent Mice, CD8+ Depleted Mice	Woller, N., et al., 2015. [20].
	MPM	N/A	Pre-Clinical Data: Athymic Mice	Kubo, S., et al., 2010. [21].
Cocksackie B3 Virus	NSCLC	Immunogenic Cell Death	Pre-Clinical Data: Immune Competent Mice	Miyamoto, S. et al., 2012. [22].
Herpes Simplex Virus	NSCLC	N/A	Pre-Clinical Data: Murine Lung Cancer Model	Goodwin et al., 2012. [23].
	NSCLC	N/A	Pre-Clinical Data: In Vitro Work	Li, J.-M., et al., 2013. [24].
	MPM	N/A	Pre-Clinical Data: Athymic Mice	Adusumilli, P.S., et al. 2006 [25].
Measles Virus	MPM	DC Maturation; CD8+ Priming	Pre-Clinical Data: In Vitro Work	Gauvrit, A., et al., 2008. [26].
	MPM	Innate Immune Infiltration by Macrophages/Monocytes	Pre-Clinical Data: Athymic Mice	Li, H., et al., 2010. [17].
	NSCLC	N/A	Pre-Clinical Data: Nude Mice	Patel, M.R., et al., 2014. [27].
Newcastle Disease Virus	NSCLC	N/A	Pre-Clinical Data: In Vitro Work	Meng, G., et al., 2014. [28].
	NSCLC	N/A	Pre-Clinical Data: In Vitro Work	Fu, F., et al., 2011. [29].
Reovirus	NSCLC	N/A	Phase I Clinical Trial w/Paclitaxel and Carboplatin	Villalona-Calero, M.A., et al., 2016. [13].
	NSCLC	N/A	Pre-Clinical Data: In Vitro Work	Sei, S., et al., 2009. [14].
	NSCLC	Cytokine Induction	Pre-Clinical Data: Mice Models	Campion, C.A., et al., 2016. [30].
	NSCLC	Increased Antigen Presentation	Pre-Clinical Mouse Model	Gujar, S.A., et al., 2010 [31].
Vaccinia Virus	NSCLC	Cytokine Expression and CD8+ Tumor Infiltration	Pre-Clinical Data: Immune Competent Mice	Wang, L.-C.S., et al., 2011. [15].
	MPM	N/A	Pre-Clinical Data: Immune Competent Mice	Acuna, S.A., et al., 2014. [32].
	MPM	N/A	Pre-Clinical Data: Athymic Mice	Belin, L.J., et al., 2013. [33].
	MPM	N/A	Pre-Clinical Data: Athymic Mice	Kelly, K.J., et al., 2008. [34].
Vesicular Stomatitis Virus	NSCLC	CD8+ Infiltration, TILs, PDL-1 Expression	Pre-Clinical Data: Immune Competent Mice	Patel et al., 2015. [8].
	MPM	Interferon Response	Pre-Clinical Data: SCID Mice	Saloura, V., et al., 2010. [9].
	MPM	CD8+ T Cell and NK Cell Infiltration	Pre-Clinical Data: Immune Competent Mice	Willmon, C.L., et al., 2009. [16].

DC = Dendritic Cell, TIL= tumor infiltrating lymphocytes, NK = Natural Killer, SCID = Severe combined immunodeficient, N/A = Not applicable.

**Table 2:** Ongoing clinical trials of oncolytic viruses for thoracic cancers.

Oncolytic Virus	Disease	Other Therapeutic Agents Involved	Status of Trial	Phase	ClinicalTrials.gov Number
Adenovirus Vaccine Expressing MAGE-A3 with Genetically-Modified MAGE-A3-Expressing MG1 Maraba Virus Vaccine	Metastatic/Advanced Non-Small Cell Lung Cancer	Pembrolizumab	Not Yet Recruiting	I/II	NCT02879760
Group B Oncolytic Adenovirus (ColoAd1)	Non-Small Cell Lung Cancer	N/A (patients must be candidates for surgery)	Completed	I	NCT02053220
Wild-Type Reovirus	Non-Small Cell Lung Cancer (must have KRAS or EGFR activation)	Carboplatin and Paclitaxel	Completed—Preliminary data indicates greatly increased median survival for patients with EGFR activation	II	NCT00861627 [13]
Thymidine Kinase-Deleted Vaccinia Virus	Metastatic/Advanced Non-Small Cell Lung Cancer	GM-CSF	Completed	I	NCT00625456
Measles Virus Vaccine Encoding Thyroidal Sodium Iodide Symporter	Malignant Pleural Mesothelioma	N/A	Recruiting	I	NCT01503177
Cocksackie A21 Virus	Non-Small Cell Lung Cancer	Pembrolizumab	Not Yet Recruiting	Ib	NCT02824965
Seneca Valley Virus (SVV-001)	Small Cell Lung Cancer	N/A	Completed—Negative trial	II	NCT01017601
Herpes Simplex Virus Type 1 (HSV-1716)	Malignant Pleural Mesothelioma	N/A	Active, Not Recruiting	I/II	NCT01721018
GL-ONC1 (Vaccinia Virus Strain)	Malignant Pleural Mesothelioma	-	Recruiting	Ib	NCT01766739
